# Supplementary figures and images for: Production of recombinant D-allulose 3-epimerase utilizing an auto-induction approach in fermentor cultures suitable for industrial application
Source: PLoS One. 2025 Jul 9;20(7):e0327420. doi: 10.1371/journal.pone.0327420 (PMC12240355; doi:10.1371/journal.pone.0327420)

**Figure 1**

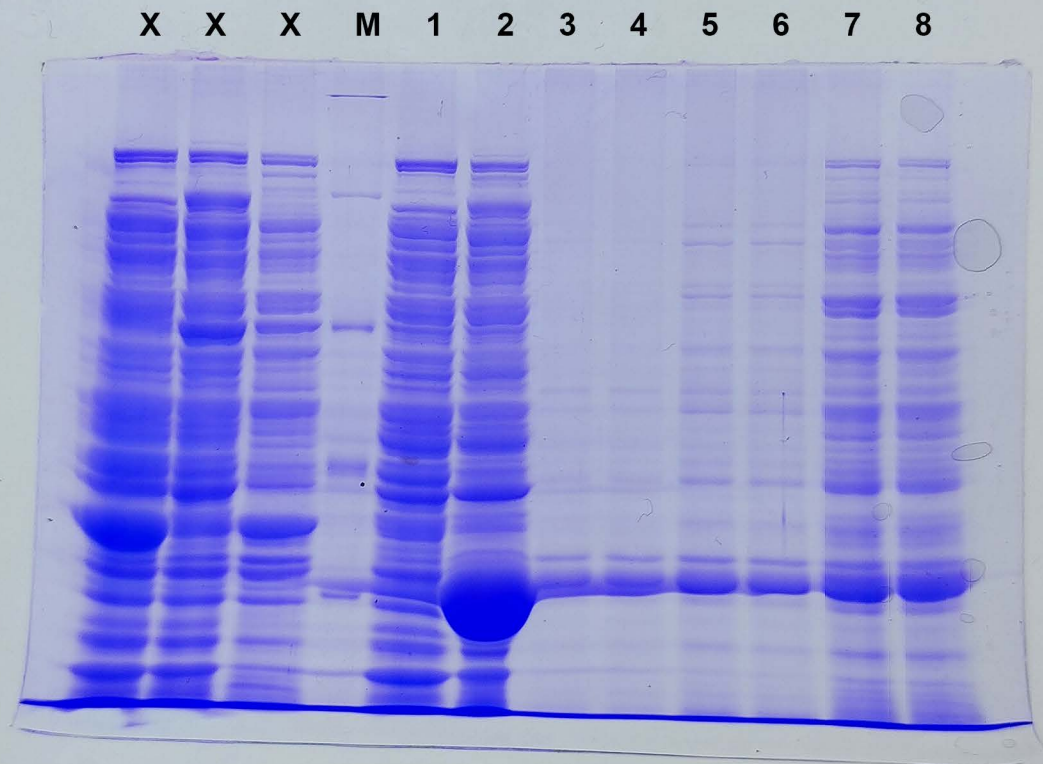

Supplement: S1 File — S1 raw images Fig 1. SDS-PAGE analysis of ApDAEase expression. The supporting files consist of unprocessed raw images of Fig 1. SDS-PAGE analysis representing the expression of Ap DAEase. Images were captured using a mobile phone positioned above a white lightbox, without any modifications in brightness, contrast, or cropping. Lane M corresponds to the protein marker; lane 1 represents the negative control; lane 2 shows the soluble fraction of Ap DAEase produced via the IPTG induction method; lanes 3 and 4 depict the soluble fraction of Ap DAEase expressed using the auto-induction method with a 72-hour incubation; lanes 5 and 6 represent the soluble fraction produced with the auto-induction method after 48 hours of incubation; and lanes 7 and 8 illustrate the soluble fraction obtained through the auto-induction method following 24 hours of incubation. Lane X indicates an unused lane. S2 raw images Fig 6A. SDS-PAGE analysis of purified ApDAEase. The supporting files consist of unprocessed raw images of Fig 6A. SDS-PAGE analysis representing the purified of Ap DAEase. Images were captured using a mobile phone positioned above a white lightbox, without any adjustments in brightness, contrast, or cropping. Lane M corresponds to the protein marker; lane 1 contains crude protein; lane 2 shows the HisTrap-purified Ap DAEase; and lane 3 represents the HiTrap-purified Ap DAEase. Lane X indicates an unused lane. S3.raw images Fig 6B. Western Blot of ApDAEase. The supporting files consist of unprocessed raw images of Fig 6B. Western blot analysis illustrating the detection of Ap DAEase. Images were captured using a mobile phone positioned above a white lightbox, without any modifications in brightness, contrast, or cropping. Lane M corresponds to the protein marker, while lane 1 represents the purified fraction of Ap DAEase. Lane X indicates an unused lane. S4 dataset Table 1. Purification Step of ApDAEase.The supporting files comprise a dataset for Table 1 presenting the puri [file pone.0327420.s001.zip › S1_raw_images_Figure 1. SDS-PAGE analysis of ApDAEase expression.pdf]

**Figure 6A**

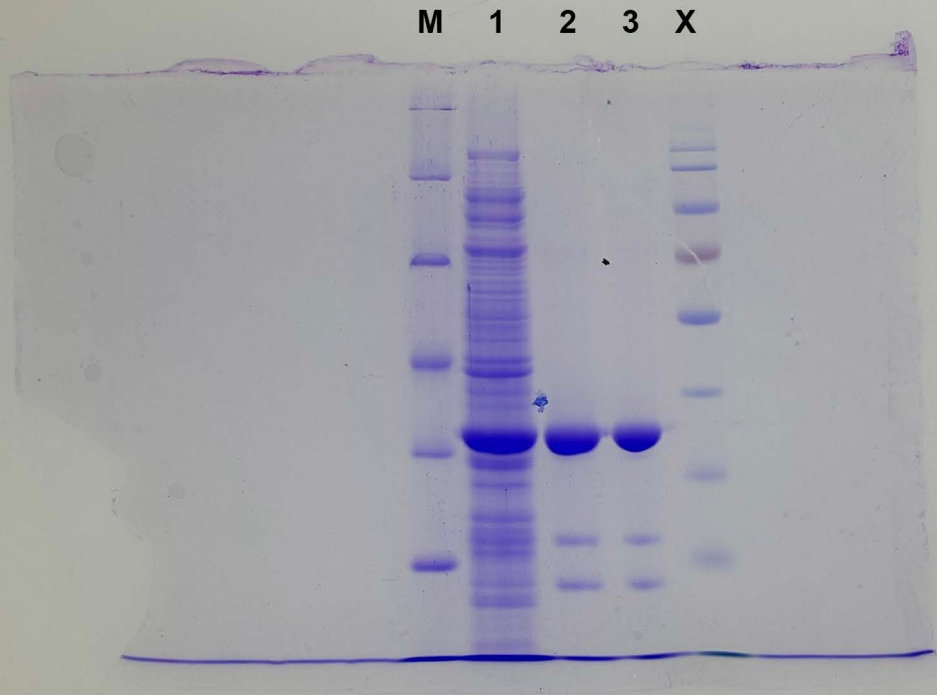

Supplement: S1 File — S1 raw images Fig 1. SDS-PAGE analysis of ApDAEase expression. The supporting files consist of unprocessed raw images of Fig 1. SDS-PAGE analysis representing the expression of Ap DAEase. Images were captured using a mobile phone positioned above a white lightbox, without any modifications in brightness, contrast, or cropping. Lane M corresponds to the protein marker; lane 1 represents the negative control; lane 2 shows the soluble fraction of Ap DAEase produced via the IPTG induction method; lanes 3 and 4 depict the soluble fraction of Ap DAEase expressed using the auto-induction method with a 72-hour incubation; lanes 5 and 6 represent the soluble fraction produced with the auto-induction method after 48 hours of incubation; and lanes 7 and 8 illustrate the soluble fraction obtained through the auto-induction method following 24 hours of incubation. Lane X indicates an unused lane. S2 raw images Fig 6A. SDS-PAGE analysis of purified ApDAEase. The supporting files consist of unprocessed raw images of Fig 6A. SDS-PAGE analysis representing the purified of Ap DAEase. Images were captured using a mobile phone positioned above a white lightbox, without any adjustments in brightness, contrast, or cropping. Lane M corresponds to the protein marker; lane 1 contains crude protein; lane 2 shows the HisTrap-purified Ap DAEase; and lane 3 represents the HiTrap-purified Ap DAEase. Lane X indicates an unused lane. S3.raw images Fig 6B. Western Blot of ApDAEase. The supporting files consist of unprocessed raw images of Fig 6B. Western blot analysis illustrating the detection of Ap DAEase. Images were captured using a mobile phone positioned above a white lightbox, without any modifications in brightness, contrast, or cropping. Lane M corresponds to the protein marker, while lane 1 represents the purified fraction of Ap DAEase. Lane X indicates an unused lane. S4 dataset Table 1. Purification Step of ApDAEase.The supporting files comprise a dataset for Table 1 presenting the puri [file pone.0327420.s001.zip › S2_raw_images_Figure 6A. SDS-PAGE analysis of purified ApDAEase.pdf]

**Figure 6B**

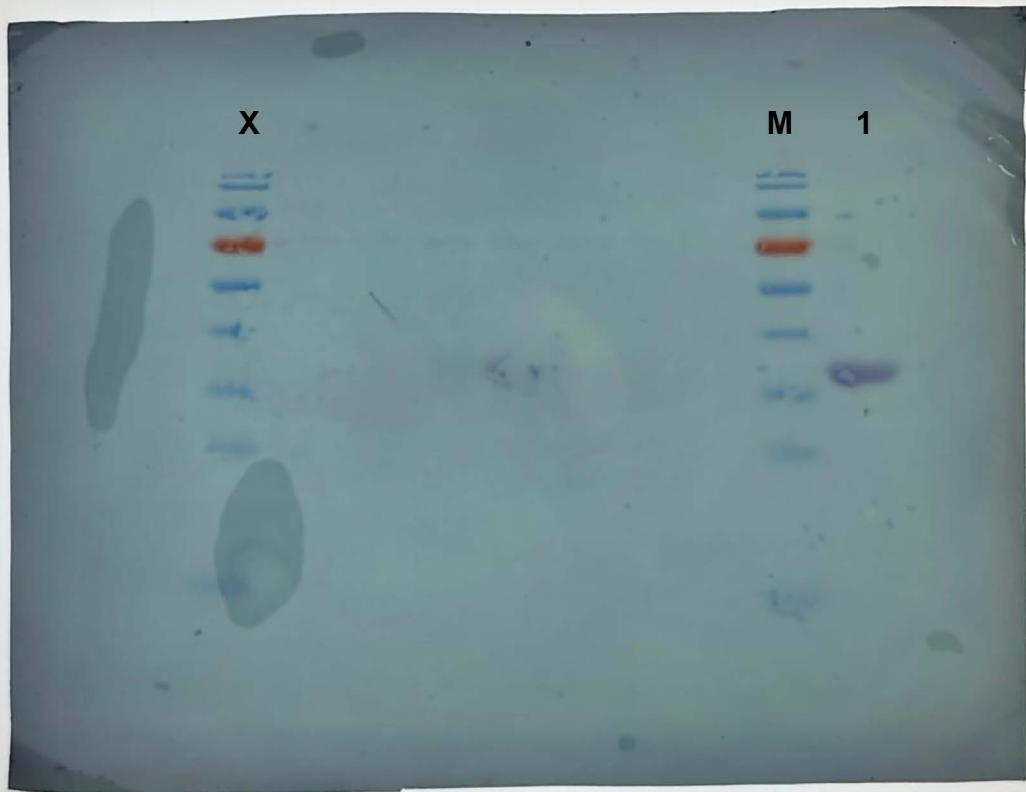

Supplement: S1 File — S1 raw images Fig 1. SDS-PAGE analysis of ApDAEase expression. The supporting files consist of unprocessed raw images of Fig 1. SDS-PAGE analysis representing the expression of Ap DAEase. Images were captured using a mobile phone positioned above a white lightbox, without any modifications in brightness, contrast, or cropping. Lane M corresponds to the protein marker; lane 1 represents the negative control; lane 2 shows the soluble fraction of Ap DAEase produced via the IPTG induction method; lanes 3 and 4 depict the soluble fraction of Ap DAEase expressed using the auto-induction method with a 72-hour incubation; lanes 5 and 6 represent the soluble fraction produced with the auto-induction method after 48 hours of incubation; and lanes 7 and 8 illustrate the soluble fraction obtained through the auto-induction method following 24 hours of incubation. Lane X indicates an unused lane. S2 raw images Fig 6A. SDS-PAGE analysis of purified ApDAEase. The supporting files consist of unprocessed raw images of Fig 6A. SDS-PAGE analysis representing the purified of Ap DAEase. Images were captured using a mobile phone positioned above a white lightbox, without any adjustments in brightness, contrast, or cropping. Lane M corresponds to the protein marker; lane 1 contains crude protein; lane 2 shows the HisTrap-purified Ap DAEase; and lane 3 represents the HiTrap-purified Ap DAEase. Lane X indicates an unused lane. S3.raw images Fig 6B. Western Blot of ApDAEase. The supporting files consist of unprocessed raw images of Fig 6B. Western blot analysis illustrating the detection of Ap DAEase. Images were captured using a mobile phone positioned above a white lightbox, without any modifications in brightness, contrast, or cropping. Lane M corresponds to the protein marker, while lane 1 represents the purified fraction of Ap DAEase. Lane X indicates an unused lane. S4 dataset Table 1. Purification Step of ApDAEase.The supporting files comprise a dataset for Table 1 presenting the puri [file pone.0327420.s001.zip › S3_raw_images_Figure 6B. Western Blot of ApDAEase.pdf]
